# Carbapenem-resistant Enterobacterales—Kentucky, 2013–2020: Challenges and Successes

**DOI:** 10.13023/jah.0503.05

**Published:** 2023-12-01

**Authors:** Mary Issac, Andrea Flinchum, Kevin Spicer

**Affiliations:** Healthcare-Associated Infection/Antibiotic Resistance Prevention Program, Division of Epidemiology and Health Planning, Kentucky Department for Public Health; Healthcare-Associated Infection/Antibiotic Resistance Prevention Program, Division of Epidemiology and Health Planning, Kentucky Department for Public Health; Healthcare-Associated Infection/Antibiotic Resistance Prevention Program, Division of Epidemiology and Health Planning, Kentucky Department for Public Health; Division of Healthcare Quality Promotion, National Center for Emerging and Zoonotic Infectious Diseases, CDC

**Keywords:** Appalachia, carbapenem-resistant Enterobacterales, CRE, healthcare-associated infections, HAI, Kentucky

## Abstract

**Introduction:**

Carbapenem-resistant Enterobacterales (CRE) are considered urgent, antibiotic-resistant threats in the U.S. and are of global concern. Active collaboration between public health authorities and healthcare facilities and providers will be necessary to prevent and contain these organisms.

**Purpose:**

To describe the epidemiology of CRE in Kentucky and to discuss challenges and successes with building and sustaining an effective prevention and containment program.

**Methods:**

Retrospective descriptive summary of CRE isolates reported by healthcare providers, facilities, and laboratories in Kentucky from 2013 through 2020. Data available from case reporting forms and laboratory testing are summarized.

**Results:**

From 2013 through 2020, 1805 CRE were reported from 1666 individuals; median age was 66 years and 44% were male. Although most reports were from hospitalized individuals, nearly one-third were from individuals not hospitalized in acute-care hospital settings. The number of reports generally increased over time, with 111 CRE isolates in 2013 and 477 in 2020. *Klebsiella pneumoniae* was the most frequently reported CRE. Of the 29% of CRE with identified carbapenemase production (CP-CRE), *Klebsiella pneumoniae* carbapenemase (KPC) was most common (78%). Surveillance and reporting resulted in identification and active investigation of 11 outbreaks of CP-CRE.

**Implications:**

There are challenges with developing, implementing, and sustaining a consistent, effective response to identifying, preventing, and containing CRE. Ongoing public health and facility resources will be necessary to prevent and contain antibiotic-resistant threats and other concerning organisms.

## INTRODUCTION

Rapid spread of carbapenem-resistant Enterobacterales (CRE) in the U.S. and globally highlights a need to actively control CRE transmission.^1^,^2^ CRE have been recognized by the Centers for Disease Control and Prevention (CDC) as an Urgent antibiotic resistance threat in the U.S.^3^ CRE that carry mobile genetic elements allowing for production of carbapenemases (enzymes than can hydrolyze β-lactam antibiotics so that they become ineffective) are of particular concern, since these mobile elements can be shared between bacteria and across bacterial species.

In 2017, the CDC outlined an initiative that encouraged healthcare facilities and public health authorities to react rapidly to novel or targeted organisms of concern, such as CRE; this containment strategy guidance was updated in 2019.^4^ Evidence suggests that these strategies—which include detection of targeted pathogens, infection-control assessment to identify gaps in infection prevention, screening of exposed contacts, coordination of response among linked facilities, and reiteration of strategies until transmission is contained— have reduced the spread of CRE nationally.^5^ However, successful implementation can be challenging for state and local health departments and healthcare facilities. These challenges include factors within both health departments and facilities, such as personnel shortages and suboptimal knowledge of organisms and how to detect, report, and respond upon identification. Challenges may be particularly impactful in jurisdictions with higher levels of poverty, fewer healthcare providers, more limited access to healthcare facilities, and a greater proportion of the population with medical comorbidities, such as in rural Kentucky and Appalachia.^6^,^7^

The first identification of the novel carbapenemase *Klebsiella pneumoniae* carbapenemase (KPC) in the U.S. was reported in 2001 from a specimen collected in North Carolina in 1996 as part of an ongoing surveillance project.^8^ Within a decade of this initial report, KPC had been identified in nearly 75% of U.S. states. KPC was first identified in Kentucky in 2008, prior to establishment of a Healthcare-Associated Infection/Antibiotic Resistance (HAI/AR) Prevention Program and a statewide process for identification and reporting of CRE. In 2013, when CDC published their initial report on “Antibiotic Resistance Threats in the US”,^9^ the Kentucky Department for Public Health (KDPH) held a statewide webinar co-hosted by CDC. The webinar provided details about the “new” threat of CRE in the U.S. and the importance of reporting these cases to the state health department. Healthcare facilities were requested to voluntarily report all CRE utilizing the CDC case definition of CRE in use at that time.^10^ Given the seriousness of CRE and the desire to achieve more complete reporting, KDPH subsequently worked to add reporting of CRE to the state’s Reportable Disease reporting regulation in 2015.^11^ All labs and facilities in Kentucky were then to manually report all CRE identified to the HAI/AR Prevention Program.

The goals of this article are to describe the epidemiology of CRE reported in Kentucky from 2013 through 2020 and to discuss some of the challenges with building and sustaining an effective reporting, prevention, and containment program.

## METHODS

### Study Design

This study is a retrospective descriptive summary of CRE identified and reported by healthcare providers and laboratories in Kentucky, which includes both description of the CRE organisms and the patients and specimen types from which they were obtained.

### Data Source

All CRE reported by healthcare providers and laboratories with specimen collection dates between January 1, 2013, and December 31, 2020, and which were consistent with the CRE definition in use at the time of report, were considered for inclusion. Reports were made using a standard multidrug-resistant organism (MDRO) reporting form that included demographic, clinical, and laboratory information.^12^ Reports of the same CRE organism from the same patient occurring during the same calendar year were counted only once if the initial identification was from a clinical specimen. If the first identification was from a surveillance specimen, a subsequent positive clinical specimen was counted as a separate event, consistent with the Council of State and Territorial Epidemiologists (CSTE) surveillance process.^13^

### Laboratory Data

Beginning in September 2017, CRE isolates were voluntarily submitted by healthcare facilities to the state laboratory for testing for the presence of genes encoding carbapenemases, including: KPC, Verona integron-encoded metallo-β-lactamase (VIM), New Delhi metallo-β-lactamase (NDM), imipenemase metallo-β-lactamase (IMP), and oxacillinase-48 (OXA-48). CRE were phenotypically identified by antibiotic susceptibility testing performed at local laboratories. To standardize reporting across the state, CDC’s revised CRE definition was implemented for case-finding and definitions for colonization and infection were utilized. The Antibiotic Resistance Laboratory Network (AR Lab Network) regional laboratory in Madison, Wisconsin, assisted the KDPH Division of Laboratory Services (DLS) with confirmation of organism identification, determination of carbapenemase production and mechanism of carbapenem resistance (if present). Prior to routine submission of isolates to the state laboratory in 2017, testing for presence of a carbapenemase was performed only as part of larger outbreak investigations. Laboratory data were matched with those from the MDRO forms using name, date of birth, and date of specimen collection.

### Study Measures

Demographic characteristics were as reported on the MDRO Reporting form for CRE cases, which were patients with CRE isolates identified. For sex: Male, Female, or Unknown if no sex provided. Age was considered in years based on date of specimen collection, and number of cases were provided by 20-year age groups. Since few patients with a race listed were not either white or black, the categories utilized were White, Black, Other (race listed [e.g., Asian/Pacific Islander, American Indian], but not White and not Black), and Unknown.

For summary reporting of the CRE isolates, location of the patient at the time of specimen collection was divided broadly into Hospitalized and Non-Hospitalized. Hospitalized was further divided into Acute Care Hospital/Critical Access Hospital, Long-Term Acute Care Hospital, and Other/Unknown. The Non-Hospitalized Category included various outpatient locations (clinics, urgent care centers, emergency departments) v. long-term care settings (i.e., nursing homes) or location unknown. The CRE were listed as *Klebsiella pneumoniae*, *Escherichia coli*, *Enterobacter cloacae*, Other Enterobacterales, or Unknown. The specific carbapenemases considered were those previously listed in the Laboratory Data above. Non-CP-CRE were those CRE which were tested and found not to harbor a carbapenemase. Unknown were those CRE that were not available for testing to determine if a carbapenemase was present. Lastly, the specimen type from which the CRE was obtained was divided into the following categories: abscess/wound, blood, respiratory tract, urine, axilla/groin/rectum, other, unknown.

### Geographic Mapping

Healthcare facilities where CRE specimens were collected were mapped by county using geographic information system (GIS) mapping, (QGIS Development Team, QGIS Geographic Information System, and Open-Source Geospatial Foundation Project, http://qgis.osgeo.org).

### Outbreaks

Outbreaks were identified and investigated when individual patients with CRE were noted to be epidemiologically linked by person, place, or time to other patients with positive CRE results. A variety of questions were asked to determine epidemiologic linkage. These included, for example, patient location within the facility, overlap of hospitalizations within the facility, sharing of staff or mobile medical equipment, and receiving similar tests or procedures. In some instances, laboratory tests, such as pulsed-field gel electrophoresis (PFGE) or whole genome sequencing (WGS), were performed as part of outbreak investigations to determine relatedness of patient isolates.

### Statistics

Frequencies and percentages were calculated for selected patient demographic variables, location of CRE isolate specimen collection, specimen source, name of organism, and type of CRE.

### Institutional Review

This project plan and activities were reviewed by CDC and KDPH and were conducted consistent with applicable federal law and CDC policy (see e.g., 45 C.F.R. part 46.102(l)(2), 21 C.F.R. part 56; 42 U.S.C. §241(d); 5 U.S.C. §552a; 44 U.S.C. §3501 et seq.). Data utilized were collected as part of routine public health surveillance and case investigation, and this project was consequently determined to be a non-research activity. The initial manuscript was reviewed and cleared (#0900f3eb81f37402) by CDC for submission.

## RESULTS

From 2013 to 2020, 1805 CRE isolates were reported in Kentucky (Table 1). The 1805 CRE isolates were obtained from 1666 individuals (CRE cases); the median age was 66 years (range: 0 to 98 years) and 732 (43.9%) were male. Of the 1805 CRE isolates, 1178 (65.3%) were collected during an inpatient hospitalization; 567 (31.4%) were collected from outpatients. Among those collected during hospitalization, most were associated with acute-care hospitals (995, 84.5%), followed by long-term acute-care hospitals (182, 15.4%). The estimated percentage of short-stay hospitals in Kentucky that reported CRE isolates varied from 14.4% (2013) to 38.1% (2020). Outpatients with CRE most commonly were living in private residences (453, 79.9%), but a number resided in nursing homes (74, 13.1%). The highest number and proportion of CRE specimens from outpatient settings in a single year were reported in 2018 (161, 41.1%). Total isolates included both clinical and surveillance cultures. Urine was the most common specimen source (n = 917, 50.8%), followed by screening sites (axilla, groin, rectum, n = 343, 19.0%) and wounds (n = 197, 10.9%).

Among the 1805 CRE identified, *Klebsiella pneumoniae* was the most common organism (n = 542, 30.0%). Five hundred twenty CRE isolates (28.8%) had a carbapenemase identified; KPC was the most common (n = 406, 78.1%), followed by VIM (n = 71, 13.7%), then NDM (n = 19, 3.7%), and OXA-48 (n = 8, 1.5%). Mechanisms were identified in both inpatient and outpatient settings. Through surveillance and reporting, in partnership with healthcare facilities in Kentucky, from 2013 through 2020, 11 outbreaks (2013: 1, 2015: 1, 2016: 1, 2017: 2, 2018: 5, 2020: 1) of carbapenemase-producing CRE (CP-CRE)—including KPC-, NDM-and VIM-producing organisms—were identified and actively investigated by KDPH.^14^,^15^ These investigations identified 94 cases of CP-CRE, which was 18% of the total CP-CRE identified from 2013 through 2020.

**Figure 1:**
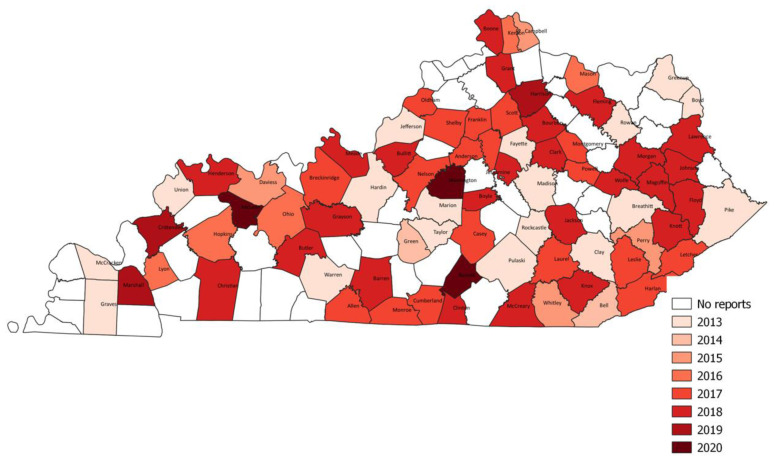
First year of carbapenem-resistant Enterobacterales (CRE) report by county, Kentucky (2013–2020) NOTE: Map shows additional counties in each year (from 2013 to 2020) that reported carbapenem-resistant Enterobacterales (CRE) when compared to previous years. Each darker shade corresponds to a successive year and illustrates counties with identification of CRE for the first time during that year.

CRE cases were reported in facilities from 78 (65%) of Kentucky’s 120 counties (Fig. 1), with 75 inpatient and 210 outpatient healthcare facilities and clinics represented. The figure illustrates the progressive detection or spread of CRE within Kentucky, with each darker shade indicating a successive year and illustrating counties with identification of CRE for the first time during that year. In 2013, facilities from 18 counties identified CRE. Over the subsequent three years (2014–2016), CRE was identified from an additional two, four, and five counties, respectively. A substantial increase in counties with cases were noted in 2017 and 2018 (18 and 25, respectively). Fewer new counties with cases of CRE were added in 2019 and during the first year of the pandemic in 2020 (three new counties each year).

## DISCUSSION

This descriptive summary of CRE reporting in Kentucky illustrates an increasing number and geographic distribution of CRE isolates from 2013 through 2020. This highlights the importance of aggressive action to prevent further dissemination of CRE. From 2013 to 2020, 11 CP-CRE outbreaks were detected, and containment principles were rapidly employed to prevent further transmission. These included on-site infection prevention assessments conducted by the HAI/AR Prevention Program, retrospective and prospective surveillance for CRE, cohorting of CRE patients where indicated, and point prevalence surveys, in addition to strengthening of infection-control measures through onsite and telephone interactions with facility infection preventionists and clinical and administrative leaders.^5^ The HAI/AR Prevention Program has increased technical assistance by phone on containment strategies, including ensuring that: (1) patients are placed into appropriate transmission-based precautions; (2) charts are flagged for use of appropriate precautions on subsequent admissions; and (3) intensive case-finding is pursued through point prevalence surveys and active surveillance. By partnering with healthcare facilities in investigating outbreaks of CRE and providing evidence-based guidelines—and in some cases using laboratory resources—these partnerships have been strengthened. As a result, surveillance and reporting of organisms have greatly improved in frequency and quality.

The HAI/AR Prevention Program surveillance has shown that CRE isolates are being identified more frequently among outpatients and nursing home residents throughout Kentucky. In response, the HAI/AR Prevention Program has emphasized more effective interfacility communication upon transfer and colonization screening for CP-CRE to limit transmission. The KDPH MDRO reporting form currently includes questions regarding whether a receiving facility was notified of a patient’s CRE status at the time of transfer. The program has also emphasized that receiving facilities should notify sending facilities if CRE is identified early in the admission as such identification likely reflects presence of the organism prior to transfer and may indicate a need for aggressive action (e.g., onsite assessment, point prevalence survey) at the sending facility.

Although the increased number of hospitals reporting CRE from 2013 through 2020 is due somewhat to the spread of CRE within the state, it may also reflect increased participation in reporting and screening by area hospitals. Following the release of the AR Threats Report in 2013, there was a period of heightened awareness and a proactive approach, including a webinar and outreach to healthcare facilities, with aggressive follow-up on any CRE reported. Reports decreased somewhat in 2014, but after the state of Kentucky mandated CRE reporting in 2015, a gradual uptick in CRE reporting was observed. Increased reporting was noted in 2017, when additional outreach was provided to facilities about identification and reporting and a request was made for voluntary submission of CRE isolates to the state public health laboratory. Although it is unclear why reported cases decreased in 2019 relative to 2018, it is possible that the impact of program activity and enhanced facility response to initial cases resulted in fewer overall CRE cases to report.

Substantial challenges remain for the achievement of complete reporting and identification of CRE. Though Kentucky mandated reporting of 10 MDROs in 2015, compliance with reporting continues to be a challenge. Facilities may have limited personnel dedicated to infection prevention activities and consequently have sub-optimal mechanisms for identification and reporting. Reporting may also vary across geographical regions within the state, as in Appalachian counties where there is a greater prevalence of poverty (household poverty rate of 26.7), higher infant mortality (infant mortality rate is 21 percent higher than nationwide rate) and increased occurrence of medical comorbidities.^6^,^7^ Geographic isolation, shortage of healthcare professionals (e.g., primary care physicians per 100,000 population is 26 percent lower than the national average), decreased access to healthcare facilities, lack of educational opportunities and other competing priorities may have also limited patient presentation, identification, and reporting of CRE.^6^,^7^ Of the 42 counties with no CRE isolates reported, 27 (64%) had no acute-care facilities within their county.

Although the number of reported isolates has generally increased year on year, there are hospitals that have not reported any CRE. It remains unclear whether this lack of reporting reflects absence of CRE in these facilities, lack of identification of CRE, or a lack of motivation to report. Electronic laboratory reporting is available through the Kentucky Health Information Exchange (KHIE),^16^ but not all laboratories have made the necessary preparations to transmit these results, given the complexity of the onboarding and coding processes necessary to ensure effective reporting. Additionally, non-electronic laboratory reporting may be suboptimal due to local staffing issues and problems with laboratory identification of CRE. However, review of the Office of Inspector General website indicates that in 2020, 62.7% of the licensed short-stay acutecare beds in the state were located within facilities that have reported CRE isolates.^17^ More recently, during the pandemic response, facilities may have forfeited reporting of MDROs given the more immediate challenge of responding to SARS-CoV-2 and the COVID-19 pandemic. However, identification of CRE isolates was higher in 2020 than in any year since reporting began.

The HAI/AR Prevention Program at KDPH is one of a number of CDC-funded programs created to increase overall national capacity to prevent and contain healthcare associated infections and antibiotic-resistant organisms, all of which may have experienced similar challenges. The challenges in recruiting experienced infection prevention and control staff, along with other competing priorities have, at times, slowed the containment response of the KDPH HAI/AR Prevention Program. Staffing issues in the state laboratory have occasionally necessitated a reliance upon the AR Lab Network regional laboratory for verification of CRE and determination of mechanisms of resistance, thereby slowing response activities. It is crucial to maintain the ability of HAI/AR Prevention Programs to quickly respond to CRE threats and aid facilities in their containment responses. Additionally, there is an ongoing need for HAI/AR Prevention Programs to have the skills and capacity to identify, investigate, and contain outbreaks of new and emerging organisms. This has been well illustrated by the nation’s challenges during the SARS-CoV-2 pandemic. Staff in many HAI/AR programs across the country, including those in the KDPH HAI/AR program, were largely diverted from MDRO and stewardship activities due to demands placed upon health departments to assist local health departments and healthcare facilities with their SARS-CoV-2 pandemic activities.^18^ The important role of the HAI/AR Prevention Program staff in education and training of frontline infection prevention and other healthcare staff also remains critical and necessary to optimize care in all healthcare settings. A national emphasis on improving knowledge and awareness of frontline healthcare workers has been established in a collaborative program involving the CDC and state, territorial, local, and tribal health departments.^19^

This descriptive summary has several limitations. First, data are not systematically reported from all microbiology labs and healthcare facilities in Kentucky to KDPH; therefore, the distribution of CRE isolates reported may not reflect the true burden of CRE across the state. Second, CRE isolates are generally from labs and facilities that have the highest compliance with the reporting mandate, which may not be representative of all facilities in Kentucky. Third, increases in reported CRE isolates may reflect an increase in cases, improvement in provider and laboratory capacity to identify and report, or a combination of these factors. Current data do not provide a means to clearly differentiate the contributions of these factors, although the higher number of CRE isolates in 2020 suggests that more recent increases reflect spread rather than improved detection, testing, and reporting. Fourth, the ongoing SARS-CoV- 2 pandemic might have impacted the occurrence and reporting of CRE in 2020. Even though the highest rate of reporting occurred in 2020, it is possible that CRE were underreported because of personnel and laboratory demands from the pandemic and CRE spread may have been even greater than suggested by the increased number of reports.^20^ Meanwhile, it has been noted that the availability and optimal use of PPE was often problematic during the pandemic, and this may have resulted in increased transmission of MDROs.^21^

## IMPLICATIONS

CRE surveillance, identification, and reporting in Kentucky have improved substantially from 2013 to 2020, as evidenced by the increased number of CRE isolates from 2013 (111) to 2020 (477). The experience in Kentucky illustrates the challenges of developing, implementing, and sustaining a consistent, effective response to identifying, preventing, and containing CRE. Additional resources, especially human resources in public health agencies and healthcare facilities, may be helpful as identification, reporting, and response burdens increase. The SARS-CoV-2 pandemic has heightened recognition of the need for improved public health infrastructure, including the critical importance of infection prevention and control. It is anticipated that with increased awareness and resources, recognition and reporting of concerning organisms will improve, as will the ability of facilities and public health to respond.

**SUMMARY BOX**

**What is already known about this topic?**

Carbapenem-resistant Enterobacterales (CRE) are urgent antibiotic resistance threats in the U.S. Strategies for containment can reduce spread and impact of these organisms, but successful implementation of a containment strategy may be difficult given competing priorities and limited resources for health departments and healthcare facilities.

**What is added by this report?**

Reports of CRE increased in Kentucky between 2013 and 2020. Although most reports were from hospitalized patients in acute care settings, an increasing number of reports were noted from the outpatient setting. Implementation of a containment strategy improved identification and reporting of CRE and strengthened health department and healthcare facility partnerships but challenges to sustaining a consistent and effective containment strategy remain.

**What are the implications for future research?**

Improved understanding of CRE reporting may enhance targeted education and intervention strategies. Further research is necessary to evaluate how reported demographic and clinical information can be utilized to improve the public health response to an identified urgent threat. Additionally, it will be necessary to continue to evaluate the impact of the public health human resource infrastructure on the ability of health departments to maintain effective partnerships and sustain containment activities.

## Figures and Tables

**Table 1:** Counts and percentages of CRE cases and isolates by year for selected characteristics

	Count (%) of CRE cases								
	2013	2014	2015	2016	2017	2018	2019	2020	Total
**Distinct patient cases** *	**105 (6.3)**	**57 (3.4)**	**81 (4.9)**	**114 (6.8)**	**196 (11.8)**	**371 (22.3)**	**301 (18.1)**	**441 (26.5)**	**1666 (100.0)**
**Sex**									
Male	49 (46.7)	22 (38.6)	32 (39.5)	57 (50.0)	86 (43.9)	144 (38.8)	146 (48.5)	196 (44.4)	732 (43.9)
Female	54 (51.4)	35 (61.4)	48 (59.3)	57 (50.0)	110 (56.1)	227 (61.2)	155 (51.5)	245 (55.6)	931 (55.9)
Unknown	2 (1.9)	0 (0.0)	1 (1.2)	0 (0.0)	0 (0.0)	0 (0.0)	0 (0.0)	0 (0.0)	3 (0.2)
**Age**									
Under 20	0 (0.0)	1 (1.8)	9 (11.1)	4 (3.5)	3 (1.5)	7 (1.9)	6 (2.0)	18 (4.1)	48 (2.9)
20–39	4 (3.8)	5 (8.8)	6 (7.4)	6 (5.3)	12 (6.1)	21 (5.7)	24 (8.0)	23 (5.2)	101 (6.1)
40–59	24 (22.9)	18 (31.6)	22 (27.2)	33 (28.9)	42 (21.4)	93 (25.1)	72 (23.9)	114 (25.9)	418 (25.1)
60–79	36 (34.3)	23 (40.4)	36 (44.4)	49 (43.0)	93 (47.4)	169 (45.6)	143 (47.5)	201 (45.6)	750 (45.0)
80–100	20 (19.0)	10 (17.5)	7 (8.6)	20 (17.5)	46 (23.5)	81 (21.8)	56 (18.6)	85 (19.3)	325 (19.5)
Unknown	21 (20.0)	0 (0.0)	1 (1.2)	2 (1.8)	0 (0.0)	0 (0.0)	0 (0.0)	0 (0.0)	24 (1.4)
**Race** †									
White	0 (0.0)	0 (0.0)	0 (0.0)	80 (70.2)	100 (51.0)	209 (56.3)	238 (79.1)	351 (79.6)	978 (58.7)
Black	0 (0.0)	0 (0.0)	0 (0.0)	6 (5.3)	10 (5.1)	21 (5.7)	15 (5.0)	19 (4.3)	71 (4.3)
Other	0 (0.0)	0 (0.0)	0 (0.0)	0 (0.0)	1 (0.5)	0 (0.0)	4 (1.3)	6 (1.4)	11 (0.7)
Unknown	105 (100.0)	57 (100.0)	81(100.0)	28 (24.6)	85 (43.4)	141 (38.0)	44 (14.6)	65 (14.7)	606 (36.4)
**Total CRE isolates** **§**	**111 (6.1)**	**60 (3.3)**	**87 (4.8)**	**127 (7.0)**	**212 (11.7)**	**392 (21.7)**	**339 (18.8)**	**477 (26.4)**	**1805 (100.0)**
**Hospitalization**									
**Hospitalized**	67 (60.4)	45 (75.0)	58 (66.7)	106 (83.5)	114 (53.8)	223 (56.9)	222 (65.5)	343 (71.9)	1178 (65.3)
Acute-care / critical access hospitals	33 (49.3)	34 (75.6)	41 (70.7)	100 (94.3)	82 (71.9)	188 (84.3)	193 (86.9)	324 (94.5)	995 (84.5)
Long-term acute care hospitals	34 (50.7)	11 (24.4)	17 (29.3)	5 (4.7)	32 (28.1)	35 (15.7)	29 (13.1)	19 (5.5)	182 (15.4)
Other / Unknown	0 (0.0)	0 (0.0)	0 (0.0)	1 (0.9)	0 (0.0)	0 (0.0)	0 (0.0)	0 (0.0)	1 (0.1)
**Non-hospitalized**	30 (27.0)	15 (25.0)	15 (17.2)	18 (14.2)	83 (39.2)	161 (41.1)	113 (33.3)	132 (27.7)	567 (31.4)
Outpatient hospitals/ clinics/ emergency departments	11 (36.7)	9 (60.0)	10 (66.7)	9 (50.0)	67 (80.7)	147 (91.3)	96 (85.0)	104 (78.8)	453 (79.9)
Long-term care/ nursing home	14 (46.7)	0 (0.0)	1 (6.7)	3 (16.7)	6 (7.2)	12 (7.5)	13 (11.5)	25 (18.9)	74 (13.1)
Other / Unknown	5 (16.7)	6 (40.0)	4 (26.7)	6 (33.3)	10 (12.0)	2 (1.2)	4 (3.5)	3 (2.3)	40 (7.1)
**Unknown**	14 (12.6)	0 (0.0)	14 (16.1)	3 (2.4)	15 (7.1)	8 (2.0)	4 (1.2)	2 (0.4)	60 (3.3)
**Organism name**									
*Klebsiella pneumoniae*	76 (68.5)	23 (38.3)	27 (31.0)	55 (43.3)	80 (37.7)	106 (27.0)	87 (25.7)	88 (18.4)	542 (30.0)
*Escherichia coli*	8 (7.2)	7 (11.7)	8 (9.2)	13 (10.2)	43 (20.3)	56 (14.3)	32 (9.4)	71 (14.9)	238 (13.2)
*Enterobacter cloacae*	16 (14.4)	15 (25.0)	34 (39.1)	24 (18.9)	55 (25.9)	98 (25.0)	92 (27.1)	174 (36.5)	508 (28.1)
Other Enterobacterales	10 (9.0)	14 (23.3)	18 (20.7)	35 (27.6)	34 (16.0)	132 (33.7)	125 (36.9)	144 (30.2)	512 (28.4)
Unknown	1 (0.9)	1 (1.7)	0 (0.0)	0 (0.0)	0 (0.0)	0 (0.0)	3 (0.9)	0 (0.0)	5 (0.3)
**Type of CRE and carbapenemase**									
**CP-CRE**	36 (32.4)	19 (31.7)	33 (37.9)	48 (37.8)	46 (21.7)	122 (31.1)	104 (30.7)	112 (23.5)	520 (28.8)
KPC	34 (94.4)	18 (94.7)	21 (63.6)	35 (72.9)	39 (84.8)	105 (86.1)	74 (71.2)	80 (71.4)	406 (78.1)
VIM	2 (5.6)	1 (5.3)	12 (36.4)	10 (20.8)	6 (13.0)	11 (9.0)	17 (16.3)	12 (10.7)	71 (13.7)
NDM	0 (0.0)	0 (0.0)	0 (0.0)	0 (0.0)	1 (2.2)	5 (4.1)	5 (4.8)	8 (7.1)	19 (3.7)
OXA	0 (0.0)	0 (0.0)	0 (0.0)	0 (0.0)	0 (0.0)	1 (0.8)	2 (1.9)	5 (4.5)	8 (1.5)
IMP	0 (0.0)	0 (0.0)	0 (0.0)	0 (0.0)	0 (0.0)	0 (0.0)	5 (4.8)	2 (1.8)	7 (1.3)
OXA and NDM	0 (0.0)	0 (0.0)	0 (0.0)	0 (0.0)	0 (0.0)	0 (0.0)	0 (0.0)	1 (0.9)	1 (0.2)
KPC and VIM	0 (0.0)	0 (0.0)	0 (0.0)	0 (0.0)	0 (0.0)	0 (0.0)	0 (0.0)	3 (2.7)	3 (0.6)
Unknown CP-CRE	0 (0.0)	0 (0.0)	0 (0.0)	3 (6.3)	0 (0.0)	0 (0.0)	1 (1.0)	1 (0.9)	5 (1.0)
**Non-CP-CRE**	0 (0.0)	0 (0.0)	0 (0.0)	6 (5.3)	40 (18.9)	175 (44.6)	138 (40.7)	289 (60.6)	642 (35.6)
**Unknown**	75 (67.6)	41 (68.3)	54 (62.1)	79 (62.2)	126 (59.4)	95 (24.2)	97 (28.6)	76 (15.9)	643 (35.6)
**Specimen Source**									
Abscess/wound	14 (12.6)	5 (8.3)	5 (5.7)	8 (6.3)	15 (7.1)	47 (12.0)	49 (14.5)	54 (11.3)	197 (10.9)
Blood	4 (3.6)	3 (5.0)	3 (3.4)	3 (2.4)	12 (5.7)	12 (3.1)	17 (5.0)	30 (6.3)	84 (4.7)
Respiratory	13 (11.7)	10 (16.7)	6 (6.9)	13 (10.2)	19 (9.0)	31 (7.9)	32 (9.4)	39 (8.2)	163 (9.0)
Urine	56 (50.4)	29 (48.3)	35 (40.2)	55 (43.3)	119 (56.1)	224 (57.1)	165 (48.7)	234 (49.1)	917 (50.8)
Axilla/groin/rectal	18 (16.2)	10 (16.7)	30 (34.5)	39 (30.7)	29 (13.7)	62 (15.8)	58 (17.1)	97 (20.3)	343 (19.0)
Other	4 (3.6)	3 (5.0)	8 (9.2)	9 (7.1)	13 (6.1)	15 (3.8)	16 (4.7)	23 (4.8)	91 (5.0)
Unknown	2 (1.8)	0 (0.0)	0 (0.0)	0 (0.0)	5 (2.4)	1 (0.3)	2 (0.6)	0 (18.0)	10 (0.6)

ABBREVIATIONS: Abbreviations: CRE = carbapenem-resistant Enterobacterales, CP-CRE = carbapenemase-producing CRE, IMP = imipenemase metallo-β-lactamase, KPC = *Klebsiella pneumoniae* carbapenemase, NDM = New Delhi metallo-β-lactamase, OXA = oxacillinase, VIM = Verona integron-encoded metallo-β-lactamase

NOTES:

* Total distinct patient cases that were identified based on date of initial test across the years from 2013 to 2020.

† Race not requested in 2013, 2014, and 2015

§ Total CRE isolates includes multiple reports from a subset of patients (In a calendar year, only one clinical specimen for a given patient with a specific organism would be included. Screening specimens are counted separately if they occur prior to a clinical specimen. Multiple organisms were identified from some individuals)
